# Effects of herb-partitioned moxibustion for ulcerative colitis: A protocol for systematic review and meta-analysis

**DOI:** 10.1097/MD.0000000000021319

**Published:** 2020-07-31

**Authors:** Xiao Yan, Fengjun Ma, Yanpu Yu, Dongqing Du, Zhilei Wang, Chen Chen, Xiaobin Zhang, Xiao Sun, Zhibin Dong, Yuxia Ma, Yuning Ma

**Affiliations:** School of Acupuncture-Tuina, Shandong University of Traditional Chinese Medicine, Jinan, Shandong, China.

**Keywords:** herb-partitioned moxibustion, meta-analysis, protocol, systematic review, ulcerative colitis

## Abstract

**Background:**

Ulcerative colitis (UC) is an idiopathic, chronic inflammatory disease of the colonic mucosa. Herb-partitioned moxibustion (HPM) treatment has been demonstrated to be effective in the treatment of UC. However, there is still a lack of high-quality evidence to support the effectiveness and safety of HPM on patients with UC. This study will aim to systematically explore the efficacy of HPM for the treatment of UC.

**Methods:**

We will search the electronic databases of Embase, MEDLINE, PubMed, Cochrane Library Central Register of Controlled Trials, China national knowledge infrastructure database (CNKI), Wan fang database, Chongqing VIP information, and SinoMed from their inception to June 2020. Randomized controlled trials (RCTs) of HPM for the treatment of UC will be included. RevMan 5.3 software (The Nordic Cochrane Center, The Cochrane Collaboration, Copenhagen, Denmark) will be applied for statistical analysis.

**Results:**

The results of this study will be published in a peer-reviewed journal.

**Conclusion:**

The conclusion of our systematic review will provide more appropriate evidence-based decisions to assist clinicians during the decision-making process when dealing with UC.

## 1 Introduction

Ulcerative colitis (UC) is a chronic inflammatory bowel disease that causes irritation, inflammation, swelling, and sores on the inner lining of the large intestine. It is clinically characterized by recurrent episodes of bloody diarrhea, cramping, and abdominal pain.^[1]^ Epidemiological surveys confirmed that the incidence of UC was reported to vary from 0.5 to 31.5 per 100,000, with a prevalence of 240 per 100,000.^[2,3]^ Moreover, the number of UC has increased in developing countries, especially in Asia.^[4]^ Studies have shown that the abnormal interaction between genetic and environmental factors play an important role in the pathogenesis of UC.^[5,6]^ However, the pathogenesis of UC has not been fully revealed, and this disease has become an important public health problem around the world. At present, the treatment of UC mainly depends on anti-inflammatory drugs, including 5-aminosalicylate compounds, corticosteroids, and immunosuppressants.^[7]^ Nevertheless, some patients who use these drugs may suffer from side effects, which may reduce health-related quality of life.^[8,9]^ Therefore, it is of significance to seek new therapies for the treatment of UC.

Traditional Chinese medicine (TCM) is a complete medical system that has been used to treat UC for a long time. As a classical method of TCM, herb-partitioned moxibustion (HPM) has been applied to treat UC in clinical practice.^[10]^ An experiment in UC rats revealed that HPM can regulate excessive local immune response by inhibiting Toll-like Receptor 2 signaling and accelerate the repair of the colonic mucosa.^[11]^ Moreover, it can promote the repair of colon injuries in UC rats by regulating the expression of several cytokines.^[12]^ With the publication of clinical trials on HPM, more and more studies have shown that HPM has good clinical effects on UC. However, there is still a lack of high-quality evidence to support the effectiveness and safety of HPM on UC. Further researches are required to synthesize and evaluate the quality of the available evidence regarding the safety and efficacy of HPM for UC. Based on this, we will systematically compare the efficacy and safety of HPM in the treatment of UC, thereby paving the way for the future treatment of UC.

## 2 Material and methods

This study has been registered at Open Science Framework (OSF, https://osf.io/). The registration DOI of this study is 10.17605/OSF.IO/7H46F. This protocol is reported following the preferred reporting items for systematic reviews and meta-analysis protocols (PRISMA-P) statement guidelines.^[13]^ If there are any changes, we will update the changes in our full review.

### 2.1 Inclusion criteria

#### 2.1.1 Study type

In this review, randomized controlled trials (RCTs) that explore the efficacy and safety of HPM in the treatment of UC will be included. Non-RCT, observational study, and experimental study will be excluded.

#### 2.1.2 Types of patients

In this work, RCT involving participants with UC will be included without restrictions of age, sex, economic status, severity of the disease, or education.

#### 2.1.3 Intervention type

In the treatment group, the interventions of the included study are HPM alone or as a combination with routine treatment recommended by guidelines. In the control group, interventions will include no treatment, placebo, and conventional pharmacotherapies recommended by guidelines. Studies with different pharmacotherapy in the control and treatment groups will be excluded.

#### 2.1.4 Primary and secondary outcomes

The primary outcome is clinical remission. Secondary outcomes included improvement of clinical symptoms, maintenance of remission, relapse rate, endoscopic remission, histological assessment, quality of life, and serious adverse events during the intervention period.

### 2.2 Search strategy

Two researchers will systematically search for eligible studies in Embase, MEDLINE, PubMed, Cochrane Library Central Register of Controlled Trials, China national knowledge infrastructure database (CNKI), Wan fang database, Chongqing VIP information, and SinoMed from their inception to June 2020. Meanwhile, we also retrieve relevant studies in Google scholar, Baidu Scholar. A search strategy that combines MeSH terms and free words will be adopted. The search strategy in PubMed is as follows:

1#: Search ((((moxibustion[MeSH Terms])) OR (herb partitioned moxibustion[Title/Abstract])) OR (herb partitioned[Title/Abstract]).
2#: (((colitis, ulcerative[MeSH Terms]) OR (colitis[MeSH Terms])) OR (ulcerative colitis[Title/Abstract])) OR (inflammatory bowel disease[Title/Abstract]).
3#: Search: (((((((((clinical trials, randomized[MeSH Terms]) OR (randomized controlled trial[MeSH Terms])) OR (controlled clinical trials, randomized[MeSH Terms])) OR (random allocation[MeSH Terms])) OR (allocation, random[MeSH Terms])) OR (controlled clinical trials, randomized[MeSH Terms])) OR (RCT[Title/Abstract])) OR (controlled clinical trial[Title/Abstract])) OR (randomized[Title/Abstract])) OR (trial[Title/Abstract]).
#1 and #2 and #3

### 2.3 Data collection and analysis

#### 2.3.1 Selection of studies

**Figure 1:**
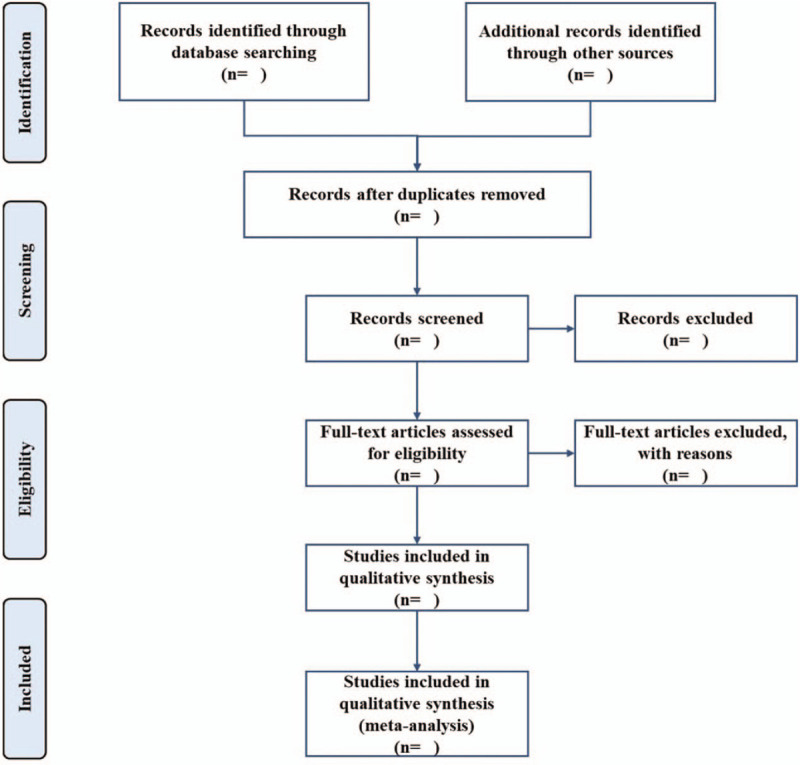
Flow chart of study selection.

The citations from the above databases will be extracted by EndNote X9.0 (Stanford, Connecticut, https://endnote.com). According to the research criteria and search strategies, 2 reviewers will review the topics and abstracts independently. The eligible articles will be further determined for inclusion by reading the full text. Any disagreements generated between the 2 reviewers will be resolved through discussion with other reviewers. A PRISMA flow chart will be drawn to illustrate the selection process (Fig. 1).

#### 2.3.2 Data extraction

Two researchers will extract information from the studies that met the inclusion criteria, including the first authors, year of publication, the severity of disease, interventions in experimental group and control group, time of treatment, ample size, age, sex, dropouts, outcome indicators, and adverse events. When data are missing, or unclear, we will contact the corresponding author for more detailed information. If the methodological details are not described in papers, we will contact the author for more explanation.

#### 2.3.3 Risk of bias assessment

In this work, Cochrane Handbook for systematic reviews of interventions Version 6 will be used to assess a broad category of biases. Two researchers will determine the bias based on the following items: random sequence generation, allocation concealment, blinding of the participants and personnel, blinding of the outcome assessments, incomplete outcome data, selective reporting, and other sources of bias. The studies will be evaluated as “Low risk,” “High risk,” or “Unclear risk.” Inconsistencies will be resolved by discussion with other reviewers.

#### 2.3.4 Data analysis

We will use the Review Manager Version 5.3 and Stata 14.0 (Stata Corporation, LLC College Station, TX, USA) software to analyze the data. The effect measure of binary variable will be calculated as risk ratio and 95% confidence interval (CIs), and measured by the mantel Haenszel method. For continuous data, we will calculate the effect size using the mean differences or standardized mean difference (SMD) with 95% CIs. The heterogeneity between the studies included in the study will be calculated by Cochrane *X*^*2*^ and *I*^*2*^ tests.^[14]^ Data will be calculated with a fixed-effect model if no statistical heterogeneity was observed (*P* ≥ .05 and *I*^2^ ≤ 50%). If *P* < .05 and *I*^*2*^ > 50%, the random effect model will be applied.

#### 2.3.5 Dealing with missing data

We will attempt to contact the corresponding author of the included studies in which there are missing data. If it fails, an intention-to-treat analysis will be performed if feasible.

#### 2.3.6 Subgroup analysis

Where possible, we will conduct subgroup analysis based on different interventions, controls, durations of treatment, and outcome measures.

#### 2.3.7 Sensitivity analysis

We will carry out sensitivity analyses to investigate the robustness of the study conclusions. The principal decision nodes include methodological quality, sample size, and the effect of missing data. Therefore, the impact of low-quality studies on the overall results will be accessed.

#### 2.3.8 Assessment of publication biases

The funnel plots in RevMan V.5.3 and Egger test in Stata 14.0 (Stata Corporation, LLC College Station, TX, USA) will be used to detect publication bias if >10 studies are included in the meta-analysis.

#### 2.3.9 Assessment of quality of evidence

The Grading of Recommendations Assessment, Development, and Evaluation (GRADE) will be used to assess the results in this systematic review. In the GRADE system, the quality of evidence can be defined as high, moderate, low, and very low.

#### 2.3.10 Ethics and dissemination

This systematic review will not require ethical approval because there are no data used in our study that are linked to individual patient data. The results will be disseminated only in a peer-reviewed publication.

## 3 Discussion

UC is a refractory, chronic, and nonspecific condition that occurs in the rectum and the entire colon. The prevalence and incidence rates of UC in Africa, Asia, and South America have increased significantly in the past decade.^[15]^ As a safe and effective external therapy in TCM, studies have shown that HPM can effectively alleviate the symptoms in patients with UC.^[16,17]^ However, there is no meta-analysis to systematically assess the clinical evidence. Therefore, we will conduct a systematic review and meta-analysis of RCT to evaluate the efficacy of HPM as a complementary and alternative medicine in the treatment of UC. The results of this review will expand our current knowledge and provide more appropriate evidence-based decisions to assist clinicians during the decision-making process when dealing with UC.

## Author contributions

**Data curation:** Xiao Yan and Fengjun Ma.

**Formal analysis:** Xiaobin Zhang and Xiao Sun.

**Methodology:** Xiao Yan, Fengjun Ma, Dongqing Du, and Zhibin Dong.

**Project administration:** Yuxia Ma.

**Resources:** Xiao Yan, Fengjun Ma, and Zhilei Wang

**Software:** Xiao Yan, Fengjun Ma, and Chen Chen.

**Visualization:** Yanpu Yu.

**Writing – original draft:** Xiao Yan and Yuxia Ma.

**Writing – review & editing:** Yuning Ma.
